# Combining COVID-19 and seasonal influenza vaccines together to increase the acceptance of newly developed vaccines in the Eastern Mediterranean Region: a cross-sectional study

**DOI:** 10.1080/07853890.2023.2286339

**Published:** 2023-11-29

**Authors:** Mohamed Fakhry Hussein, Abdelhamid Elshabrawy, Sarah Assem Ibrahim, Suzan Abdel-Rahman, Hoda Ali Ahmed Shiba, Ehab Elrewany, Mohammad Haroon Hairan, Ramy Mohamed Ghazy

**Affiliations:** a Occupational Health and Industrial Medicine Department, High Institute of Public Health, Alexandria University, Alexandria, Egypt; b Biostatistics and Demography Department, Faculty of Graduate Studies for Statistical Research, Cairo University, Giza, Egypt; c Public Health and Community Medicine at Faculty of Medicine (Girls), Al-Azhar University, Cairo, Egypt; d Tropical Health Department, High Institute of Public Health, Alexandria University, Alexandria, Egypt; e Department of Natural Resources Management, Faculty of Environment, Kabul University, Kabul, Afghanistan

**Keywords:** COVID-19, vaccine hesitancy, seasonal influenza vaccine, health promotion, Eastern Mediterranean Region

## Abstract

**Background and aim:**

The World Health Organization (WHO) recommended the concomitant administration (co-administration) of inactivated seasonal influenza and coronavirus disease 2019 (COVID-19) vaccines, encouraging the practice for the 2021–2022 flu season. This study aimed to assess the acceptance of simultaneously receiving the seasonal influenza vaccine (SIV) and the COVID-19 vaccine in a single administration to reduce vaccine rejection towards the COVID-19 vaccination.

**Methods:**

An online-based cross-section survey was conducted from 1 September to 9 November 2022, in the Eastern Mediterranean Region (EMR) through distributing the survey on different social media platforms, including Facebook, Twitter, LinkedIn and WhatsApp. We used the multi-level model to assess the variation of vaccine countries across EMR countries.

**Results:**

In total, 3300 participants were included in this survey from 11 countries distributed in the EMR. More than one-third (40.7%) were aged 18–25 years, 60.6% were females, 54.0% had a university degree, 43.1% had previous COVID-19, and 41.9% had relatives or friends who died from COVID-19. In total, 43.3% accepted this combination because it is less costly (9%), safer (18%), more effective (17%), and has fewer doses (19%). Rejection of this combination was due to fear of side effects (31%), and no studies have been published on their effects (31%). There was a significant difference across countries, which accounted for 6% of the variance in the log-odds of accepting the combined vaccination. Multi-level analysis revealed that being male, African and losing a family member or friend from COVID-19 increased the acceptance of the theoretical combined vaccines. Additionally, the number of doses taken of the COVID-19 and influenza vaccines separately significantly affected the combined vaccine acceptance. However, previous COVID-19 infection and older age reduced the odds of accepting the combined vaccines. Occupational level, social status and educational level didn’t significantly affect the acceptance odds.

**Conclusions:**

We can conclude that combining SIV and COVID-19 vaccines in one shot increased the overall acceptance of COVID-19 vaccines among vaccine rejectors.

## Introduction

The coronavirus disease 2019 (COVID-19) pandemic has had a significant impact globally, leading to high morbidity and mortality rates. As of 24 May 2023, the World Health Organization (WHO) reported approximately 766 million confirmed cases and 6.9 million deaths worldwide. These figures highlight the widespread nature and severity of the disease. In the Eastern Mediterranean Region (EMR), which includes countries in the Middle East and North Africa, there have been approximately 23 million confirmed cases and 350 thousand deaths attributed to COVID-19 [1]. Approximately 13 billion doses of COVID-19 vaccines have been administered worldwide. This indicates a significant global effort to vaccinate populations against the virus. According to the given statistics, around 68.2% of the world population has received at least one dose of the vaccine [2].

Seasonal influenza is an acute respiratory infection caused by types A and B influenza viruses. It is known to cause seasonal epidemics, which typically occur during the winter months in temperate climates. However, in tropical regions, influenza can occur throughout the year due to less pronounced seasonal variations [3]. It is estimated that seasonal influenza may result in up to 650,000 deaths each year due to respiratory diseases [4]. To mitigate the impact of seasonal influenza, the WHO recommends annual vaccination with the seasonal influenza vaccine (SIV). This vaccine is designed to provide protection against the prevalent strains of influenza viruses circulating in a given season. This vulnerable group includes health workers, pregnant women, children, the elderly and people living with chronic conditions [5]. According to the Centers for Disease Control and Prevention (CDC), SIV coverage has decreased in recent years. In the 2019–2020 season, the SIV coverage was reported to be 63.7% among the population. However, in the following seasons, there was a decrease in coverage, with rates of 58.6% in 2020–2021 and 57.8% in 2021–2022, which was mainly due to the change in population concern towards the COVID-19 pandemic [6,7].

As of October 2022, the Global Influenza Surveillance and Response System (GISRS) reported that during the COVID-19 pandemic, there had been a notable decrease in the global detection of influenza viruses. This decline is primarily attributed to the various measures implemented worldwide to prevent and control the spread of COVID-19, such as mask-wearing, physical distancing and enhanced hygiene practices [8]. The latest available data suggest low global activity for influenza. Additionally, among the detected cases, type A influenza viruses have been more prevalent [9].

In autumn 2021, the WHO recommended the concomitant administration (co-administration) of inactivated seasonal influenza and COVID-19 vaccines, encouraging the practice for the 2021–2022 flu season. This approach involves administering the two vaccines simultaneously but at separate anatomical sites, such as different arms [10]. Administering both vaccines during the same visit offers multiple benefits. At the individual level, it reduces the required healthcare visits and ensures timely protection against both diseases. This convenience encourages higher uptake of the two vaccines as individuals can conveniently receive them together. On a health system level, co-administration streamlines the implementation of both vaccine programs and alleviates the burden on healthcare services. By combining the administration of vaccines, healthcare providers can efficiently manage resources and optimize their vaccination efforts [10]. The co-administration of seasonal inactivated influenza and COVID-19 vaccines was also supported by the co-circulation of influenza viruses and severe acute respiratory syndrome coronavirus 2 (SARS-CoV-2), the causative agent of COVID-19 [11]. The administration of the SIV has been associated with several benefits in relation to COVID-19. It has been observed that receiving the SIV can reduce the severity of COVID-19 symptoms, decrease the requirement for intensive care, decrease the need for ventilator support, and lower mortality rates [12,13].

Fortunately, a theoretical combination of SIV and COVID-19 vaccines in one shot has been suggested to increase the acceptance rate of COVID-19 vaccination. Many pharmaceutical companies have started to develop this new modality of vaccination, and various clinical trials that have been carried out have shown initial results of the high safety and efficacy of this combination [14–16].

From the point of view of vaccine hesitancy, which could be described as the reluctance or refusal to be vaccinated despite the availability of vaccines [17], vaccine co-administration or combination vaccines may encounter additional challenges, owing to misconceptions regarding their efficacy and safety. Specifically, laypeople may believe that too many vaccines or antigens overload the immune system, may be less effective than the same vaccines administered alone, or may be more reactogenic [18].

To design effective targeted health promotion interventions related to the co-administration or combination of COVID-19 and SIV, it is valuable to assess the level of public acceptance and its determinants. Quantifying public acceptance involves understanding individuals’ willingness to receive both vaccines during the same visit and their attitudes towards the combined approach. In an Italian survey, approximately two-thirds of the participants indicated a willingness to receive both COVID-19 vaccine and SIV in one shot. Several factors were found to influence positive attitudes towards vaccine co-administration significantly. These included adherence to the primary COVID-19 vaccination schedule, prior receipt of SIV, trust in public health institutions, being male, younger age, willingness to pay for SIV out-of-pocket, perceived severity of influenza and recent information-seeking behaviour related to influenza [19].

Developing any health measure requires community acceptance; otherwise, it would be ineffective. Being a major public health concern, it is crucial to address factors that may influence the acceptance of vaccination, presenting the COVID-19 vaccine as a model. The present study aimed to assess the acceptance of simultaneously receiving the SIV and the COVID-19 vaccine in a single shot, with the goal of reducing vaccine rejection towards the COVID-19 vaccination in the EMR. It could help to explore the incentives that could be introduced to the population to increase the acceptance of vaccines in routine and epidemic situations in the future.

## Methodology

### Study design and setting

An online-based cross-section survey was conducted from 1 September to 9 November 2022, in 11 of the 22 countries of the EMR (Egypt, Iraq, Kuwait, Lebanon, Libya, Morocco, Pakistan, Afghanistan, Sudan, Saudi Arabia and Yemen) through the distribution of the survey *via* social media platforms (Facebook, Twitter, LinkedIn and WhatsApp).

### Study population

Participants were recruited if they were 18 years old or older, could read English or Arabic, had an online account with access to the internet *via* computer or smartphone, and were residents in one of the EMR countries during the COVID-19 pandemic.

### Sampling methods and sample size

A non-random sampling design was used (convenience and snowball sampling techniques) to collect the required sample sizes. According to Maas and Hox [20] and Moineddin et al. [21], to achieve a balance between the number of observations drawn from different countries, we designed the multi-level logistic regression model to be satisfied with the first 300 observations from each country. Eleven countries were included from Africa (four countries) and Asia (seven countries), with 330 participants per country, ending up with 3300 participants (1200 Africans and 2100 Asian participants).

### Data collection tool

To assess the accessibility and practicality of the online survey, a pilot study was conducted. Each collaborator was asked to collect three responses using the online questionnaire. The pilot study revealed that it took participants approximately 5–15 min to complete the questionnaire (see Supplementary file, Study questionnaire covid infl MENA). Some minor rephrasing of certain words was necessary to enhance clarity.

An anonymous questionnaire was created and uploaded to a Google Form in two languages: English and Arabic. The questionnaire consisted of four sections. The first section collected socio-demographic data (i.e. age, sex, education, social status and occupation). According to the International Labour Organization, the four categories of occupations were: high-skilled (non-manual) occupations, including managers, professionals, legislators, senior officials, technicians and associate professionals; low-skilled (non-manual) occupations, including service workers, clerks and market sales workers; skilled manual occupations, including craft and related trades workers, skilled agriculture and fishery workers, plant and machine operators and assemblers; and others, including elementary workers, students and non-working participants [22] (see Supplementary Material, Job Categories).

The second section was developed to obtain data about health conditions (history of chronic disease ‘conditions that last 1 year or more and need continuous medical supervision or limit activities of daily living or both. Chronic diseases such as heart disease, cancer, renal insufficiency, liver insufficiency, neurological diseases, and diabetes’ [23], previous COVID-19 infection, and COVID-19 deaths among relatives and friends) and the attitude towards the COVID-19 vaccine (I took the first, second and booster doses, I was waiting for the booster dose, I was waiting for the second dose, I would not take the booster dose, I took the first dose but would not take any other doses and I didn’t take any doses).

The third section also gathered data about the attitude towards the SIV (I took the vaccination last year and current year, took vaccination last year and I was waiting for the vaccine this year, did not get vaccinated before but would take it this year, I took it last year but would not take it this year, I did not take the vaccination and I would not take it this year).

The fourth section asked participants who were not vaccinated against COVID-19 about their attitude towards the COVID-19 vaccine if it was given in one dose with the influenza vaccine. Then acceptors and rejectors of the hypothetical mixture were asked about the causes of approval or refusal. All respondents were restricted to submitting only one response to ensure the accuracy of the data.

### Studied variables and outcome

This study aims to identify the main drivers responsible for accepting COVID-19 and seasonal influenza vaccinations in one dose using the multi-level logistic regression model. The dependent variable was a binary variable that took 1 in the case of accepting the two vaccinations in one dose and 0 otherwise. While the independent variables were analysed on two levels, the first level includes socio-demographic variables such as age, gender, educational status, marital status and occupation: health variables such as having chronic diseases, contracting COVID-19 before, experiencing the death of a relative or friend with COVID infection, and vaccination status for seasonal influenza, vaccination status for COVID-19. The second level includes the country of residence and continent (Asia, Africa).

### Ethical considerations

This study was approved by the Ethical Committee of the Faculty of Medicine, Alexandria University, Egypt (IRB No. 00012098/FWA No. 00018699). The study was executed according to the ethical standards laid down in the 1964 Declaration of Helsinki and its later modifications or comparable ethical standards [24]. The purpose of the research was stated at the beginning of the questionnaire, and participants were able to accept or reject participation. They were able to withdraw from the survey at any time before its completion. Participants were assured that all data would be used only for research purposes. Participants’ answers were anonymous and confidential. Responses were saved only by clicking on the provided ‘submit’ button.

### Statistical analysis

The data were gathered and pooled into an Excel spreadsheet. Statistical analysis was carried out using the R package called lme4 [25]. Categorical variables were presented as frequency and percentage. The chi-square (χ^2^) test of independence was used to test the association between the dependent variables (acceptance or rejection of taking both vaccines together in one shot) and the independent variables such as age, gender, educational level, occupation, suffering from a chronic disease and previous SARS-CoV-2 infection. A *p*-value <.05 was considered to be statistically significant for inferential analysis, if applicable. Figures were designed using Microsoft PowerPoint and the ggplot2 program.

### Statistical model

We conducted a multi-level analysis to account for the variability across countries [26]. Multi-level analysis is suitable for clustering the data avoiding underestimating the parameters. The model takes the following form:

$$\mathrm{Logit}(\frac{Y_{ij}}{1-Y_{ij}})=\beta_{00}+(\beta_{10}+U_{1j})Z_{ij}+\beta_{01}Z_{j}+U_{0j}.$$

The proportion ($P(Y_{ij}=1$) to (1–$P(Y_{ij}=1))$ is the odds of accepting COVID-19 and seasonal influenza vaccinations in one dose. $\beta_{00}\;$ is the fixed intercept, and $U_{0j}$ is the random effect of the intercept. The random effect has a normal distribution with variance $\sigma_{uo}^{2}$. $Z_{ij}$ includes the first level independent variable, $\beta_{10}$ is the fixed slope, $U_{1j}$ is the country-specific slope deviation for specific variables, $Z_{j}$ are country-level variables, $\beta_{01}$ is the fixed slope of country-level variables. The AIC (Akaike information criterion), BIC (Bayesian information criterion), and the likelihood ratio test were measured to assess the goodness of fit of the model [27]. We used the log-likelihood ratio test to justify the importance of adding a random slope of some independent variables to the model and the intraclass correlation coefficient (ICC) to assess the model’s performance. The model was conducted using R program based on the lme4 package used to estimate the model [25].

## Results

### Socio-demographic characteristics of the participants and the information related to SARS-CoV-2 infection

A total of 3300 participants were included in this survey from 11 countries distributed in the EMR, with equally distributed observations with 300 respondents from each country. More than two-fifths (40.7%) were aged 18–25 years old, (60.6%) were females, more than half (54.0%) had a university degree, 55.1% were unmarried, 38.3% had a high-skilled (non-manual) job, 43.1% of them had previous SARS-CoV-2 infection, around two-fifths of them (41.9%) had relatives or friends died from SARS-CoV-2 infection, and nearly three-quarters (72.7%) of the respondents were from Asia. Approximately half of the participants would take the COVID-19 and seasonal influenza vaccinations, if available, in one shot (Table 1).

**Table 1.** Baseline characteristics of respondents (*N* = 3300).

Variable	*n* (%)
Age categories	
18 to <25 years	1343 (40.7%)
25 to <35 years	925 (28.0%)
35 to <50 years	671 (20.3%)
50 to 65 years	249 (7.6%)
Above 65 years	112 (3.4%)
Gender	
Female	1998 (60.6%)
Male	1302 (39.4%)
Level of education completed	
Primary education	47 (1.4%)
Secondary education	659 (20.0%)
University education	1782 (54.0%)
Postgraduate	812 (24.6%)
Social status	
Unmarried	1817 (55.1%)
Married	1483 (44.9%)
Occupation	
High-skilled (non-manual)	1263 (38.3%)
Low-skilled (non-manual)	299 (9.1%)
Skilled (manual)	77 (2.3%)
Others	1661 (50.3%)
Suffering from chronic diseases	
No	2664 (80.7%)
Yes	636 (19.3%)
Had previous COVID-19 infection	
No	1345 (40.8%)
Yes	1421 (43.1%)
I don’t know	534 (16.1%)
Had relatives or friends died from COVID-19 infection	
No	1644 (49.8%)
Yes	1384 (41.9%)
Maybe	272 (8.3%)
Continent	
Asia	2400 (72.7%)
Africa	900 (27.3%)
Intention of taking COVID and influenza vaccines together in one shot	
I will take this combined vaccine	1428 (43.3%)
I will not take this combined vaccine	1872 (56.7%)

### Distribution of respondents according to their COVID-19 and influenza vaccine status

**Figure 1.**
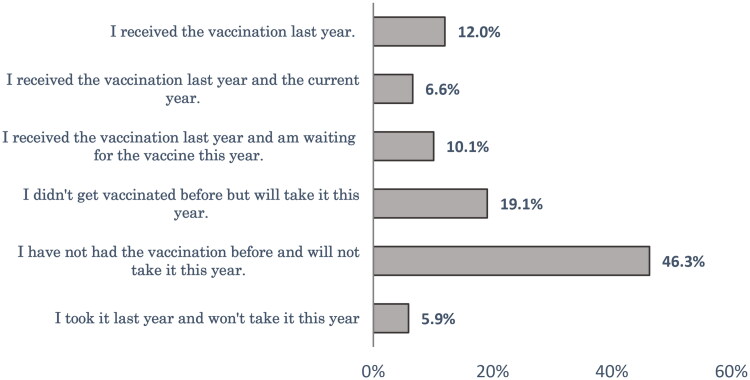
Status of the respondents regarding seasonal influenza vaccine.

**Figure 2.**
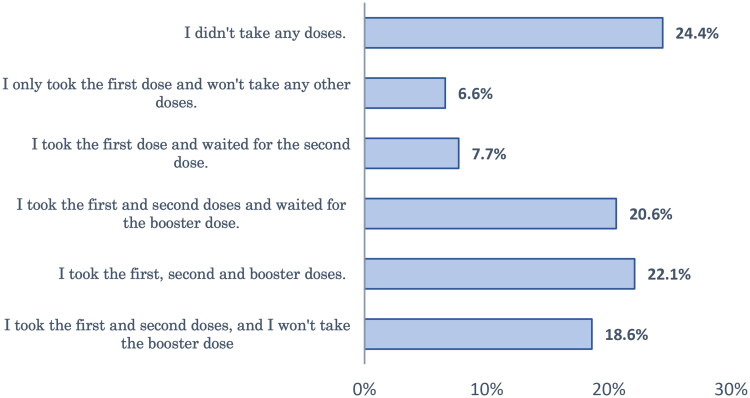
Status of the respondents regarding COVID-19 vaccination.

Forty-six percent of respondents had not taken SIV before and did not intend to take it this year. About one-fifth (19.1%) of the studied group had not received SIV before but were willing to get the vaccine this year (Figure 1). Regarding the COVID-19 vaccination, about one-quarter (24.4%) of the respondents didn’t take any doses, and 28% of the studied sample was waiting for the second or booster dose (Figure 2).

### Causes of accepting or rejecting the theoretical combination (COVID-19 and influenza vaccines) in one dose among participants

**Figure 3.**
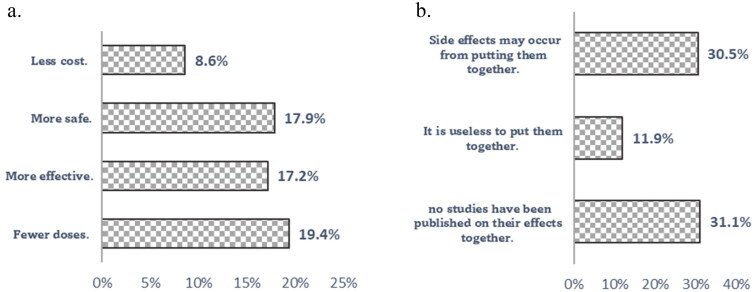
(a) Reasons for accepting the theoretical combination (COVID-19 and influenza vaccines) in one dose among participants. (b) Reasons for rejecting the theoretical combination (COVID-19 and influenza vaccines) in one dose among participants.

As shown in Figure 3, respondents preferred taking both vaccines (COVID-19 and SIV together) in one shot because it would be less costly (9%), safer (18%), more effective (17%) and with fewer doses (19%). While respondents who preferred to take the two vaccines separately indicated that side effects might occur from putting them together (31%), no studies have been published on their effects (31%).

### Respondents’ intention to take COVID-19 and seasonal influenza vaccines if they are available in one shot

As indicated in Table 2, more than two-thirds of respondents in the different age groups accepted receiving the COVID-19 and seasonal influenza vaccinations if they were available in one dose. The highest acceptance rate was in the age groups (18–24 years) and (above 65 years; 45.8% and 45.5%, respectively). Males were more likely to accept taking the two vaccinations in one dose compared to females (50% versus 39%). The higher the educational level, the greater the acceptance rate of receiving the two vaccinations in one shot. All of these differences were statistically significant (*p* < .05).

**Table 2.** Respondents’ intention to take COVID-19 and seasonal influenza vaccines if they are available in one shot.

Variable	Accepting both together	Rejecting both together	χ^2^ test and *p*-value
Age categories			
18–24 years	615 (45.8)	728 (54.2)	χ^2^ = 11.32
25–34 years	404 (43.7)	521 (56.3)	*p* = .023
35–49 years	266 (39.6)	405 (60.4)	
50–65 years	92 (36.9)	157 (63.1)	
Above 65 years	51 (45.5)	61 (54.5)	
Gender			
Female	781 (39.1)	1217 (60.9)	χ^2^ = 36.10
Male	647 (49.7)	655 (50.3)	*p* < .001
Level of education completed			
Primary education	17 (36.2)	30 (63.8)	χ^2^ = 10.14
Secondary education	252 (38.2)	407 (61.8)	*p* = .017
University education	790 (44.3)	992 (55.7)	
Postgraduate	369 (45.4)	443 (54.6)	
Country			
Egypt	144 (48.0)	156 (52.0)	
Sudan	158 (52.7)	142 (47.3)	
Kuwait	89 (29.7)	211 (70.3)	
Saudi Arabia	99 (33.0)	201 (67.0)	χ^2^ = 219.46
Morocco	180 (60.0)	120 (40.0)	*p* < .001
Iraq	113 (37.7)	187 (62.3)	
Yemen	119 (39.7)	181 (60.3)	
Lebanon	67 (22.3)	233 (77.7)	
Libya	106 (35.3)	194 (64.7)	
Afghanistan	157 (52.3)	143 (47.7)	
Pakistan	196 (65.3)	104 (34.7)	
Marital status			χ^2^ = 14.95
Unmarried	841 (46.3)	976 (53.7)	*p* = <.001
Married	587 (39.6)	896 (60.4)	
Occupation			
High-skilled (non-manual)	550 (43.5)	713 (56.5)	χ^2^ = 2.11
Low-skilled (non-manual)	118 (39.5)	181 (60.5)	*p* = .551
Skilled (manual)	32 (41.6)	45 (58.4)	
Others	728 (43.8)	933 (56.2)	
Suffering from chronic diseases			χ^2^ = 12.63
No	1112 (41.8)	1552 (58.2)	*p* < .001
Yes	315 (49.5)	321 (50.5)	
Had previous COVID-19 infection			
No	633 (47.1)	712 (52.9)	χ^2^ = 13.94
Yes	570 (40.1)	851 (59.9)	*p* = .001
I don’t know	225 (42.1)	309 (57.9)	
Had relatives or friends died from COVID-19 infection			
No	684 (41.6)	960 (58.4)	χ^2^ = 0.14
Yes	618 (44.7)	766 (55.3)	*p* = .138
Maybe	126 (46.3)	146 (53.7)	
Continent			χ^2^ = 6.19
Asia	1007 (42.0)	1393 (58.0)	*p* = .014
Africa	421 (46.8)	479 (53.2)	
For seasonal influenza vaccination			
I received the vaccination last year	196 (49.6)	199 (50.4)	
I received the vaccination last year and the current year	123 (56.2)	96 (43.8)	χ^2^ = 188.38
I received the vaccination last year and waiting for the vaccine this year	203 (61.0)	130 (39.0)	*p* < .001
I didn’t get vaccinated before but will take it this year	350 (55.6)	279 (44.4)	
I have not had the vaccination before and will not take it this year	495 (32.4)	1034 (67.6)	
I took it last year and won’t take it this year	61 (31.3)	134 (68.7)	
Uptake of the COVID-19 vaccination			
I didn’t take any doses	222 (27.5)	584 (72.5)	χ^2^ = 211.48
I only took the first dose and won’t take any other doses	98 (44.7)	121 (55.3)	*p* < .001
I took the first dose and am waiting for the second dose	125 (49.4)	128 (50.6)	
I took the first and second doses and am waiting for the booster dose	408 (60.2)	270 (39.8)	
I took the first, second and booster doses	374 (51.3)	355 (48.7)	
I took the first and second doses and I won’t take the booster dose	201 (32.7)	414 (67.3)	

Respondents with chronic diseases and those who had previous COVID-19 infection were more likely to reject the combination than those without chronic diseases or who did not have COVID-19 infection before, with a statistically significant difference (*p* < .001). More than half of the respondents who intended to get the SIV this year preferred to receive the COVID-19 and SIV in the same shot, if available. This result had a statistically significant difference (χ^2^ = 188.38, *p* < .001).

**Figure 4.**
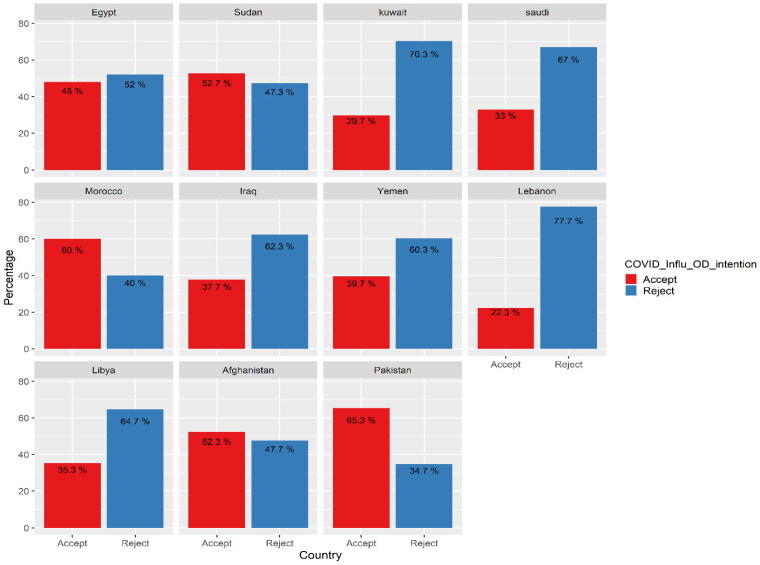
Distribution of respondents according to their intention to take the vaccination (COVID-19 and seasonal influenza) if they are available in one shot across countries.

Acceptance rates across countries show interesting findings; there was a significant difference between acceptance rates for receiving both vaccines in one shot across countries. For example, more than half of Morocco, Sudan, Afghanistan and Pakistan respondents accepted the two vaccinations in one shot. At the same time, the acceptance rate dropped to 30% in Kuwait and 22% in Lebanon. These results showed a statistically significant difference (χ^2^ = 219.46, *p* < .001; Table 2, Figure 4).

### Factors that determine acceptance of the theoretical combination (COVID-19 and seasonal influenza vaccines) in one shot among participants through a multi-level logistic model

Table 3 shows that the variation between countries accounted for 6% of the variance in the log-odds of two-dose vaccination acceptance. Hence, we utilized the multi-level logistic model to address this issue. The probability of taking two vaccinations together was 57%. The acceptance of the COVID-19 and influenza vaccines together in one shot showed more reduction with increasing participants’ age. The likelihood of the respondents’ acceptance of the theoretical combination lowered by 45% for the age group 50–65 years compared to respondents between 18 and 24 years old (odds ratio [OR] = 0.55, 95% confidence interval [CI] [0.39–0.80], *p* < .01).

**Table 3.** The results of multi-level logistic model with random intercept and a random slope.

Variable	Random intercept model	Random intercept and random slope
	Odds ratio [95% CI]	Odds ratio [95% CI]
Intercept	0.32** [0.14–0.74]	0.29** [0.14–0.63]
Age categories		
18–24 years (reference group)		
25–34 years	0.99 [0.79–1.24]	0.97 [0.77–1.21]
35–49 years	0.81 [0.62–1.07]	0.84 [0.64–1.10]
50–65 years	0.54** [0.38–0.78]	0.55** [0.39–0.80]
Above 65 years	0.50** [0.38–0.78]	0.50** [0.31–0.79]
Gender		
Female (reference group)		
Male	1.18 [1.00–1.39]	1.21* [1.03–1.42]
Education level		
Primary education (reference group)		
Secondary education	1.11 [0.56–2.20]	1.25 [0.64–2.47]
University education	1.14 [0.58–2.24]	1.28 [0.66–2.49]
Postgraduate	1.14 [057–2.27]	1.35 [0.68–2.66]
Marital status		
Unmarried (reference group)		
Married	0.92 [0.76–1.11]	0.91 [0.75–1.10]
Occupation		
High-skilled (non-manual; reference group)		
Low-skilled (non-manual)	0.83 [0.62–1.12]	0.83 [0.62–1.12]
Skilled (manual)	0.86 [0.51–1.47]	0.85 [0.50–1.44]
Others	1.02 [0.83–1.26]	1.04 [0.84–1.27]
Suffering from chronic diseases		
No (reference group)		
Yes	1.17 [0.95–1.44]	1.14 [0.92–1.41]
Having COVID-19 infection before		
No (reference group)		
Yes	0.82* [0.68–0.98]	0.79* [0.65–0.95]
I don’t know	0.98 [0.79–1.23]	0.94 [0.75–1.18]
Any family members or friend died of COVID-19 infection		
No (reference group)		
Yes	1.24* [1.05–1.47]	1.22* [1.03–1.44]
Maybe	1.12 [0.84–1.49]	1.11 [0.83–1.48]
Continent		
Asia (reference group)		
Africa	1.36 [0.73–2.54]	1.55* [1.10–2.17]
For seasonal influenza vaccination		
I received the vaccination last year. (Reference group)		
I received the vaccination last year and the current year	1.39 [0.97–1.99]	1.60* [1.03–2.48]
I received the vaccination last year and waiting for the vaccine this year	1.53** [1.11–2.10]	1.52* [1.09–2.13]
I didn’t get vaccinated before but will take it this year	1.14 [0.87–1.50]	1.23 [0.86–1.75]
I have not had the vaccination before and will not take it this year	0.55*** [0.43–0.71]	0.59* [0.36–0.98]
I took it last year and won’t take it this year	0.50*** [0.34–0.75]	0.51** [0.33–0.79]
Uptake of the COVID-19 vaccination		
I didn’t take any doses (reference group)		
I only took the first dose and won’t take any other doses	2.55** [1.79–3.63]	2.29*** [1.62–3.25]
I took the first dose and am waiting for the second dose	2.56*** [1.75–3.73]	2.09*** [1.45–2.99]
I took the first and second doses and am waiting for the booster dose	4.50*** [3.39–5.98]	4.01*** [3.04–5.28]
I took the first, second and booster doses	4.11*** [3.03–5.57]	3.49*** [2.61–4.66]
I took the first and second doses, and I won’t take the booster dose	2.03*** [1.51–2.74]	1.85*** [1.38–2.48]
Random effects		3.29
σ_2_	3.29	0.00_country_
σ_00_	0.20_country_	0.15_Country.Influ_vaccinationI received the vaccination last year and the current year_
σ_11_		0.05_Country.Influ_vaccinationI received the vaccination last year and am waiting for the vaccine this year_
		0.16_Country.Influ_vaccinationI didn’t get vaccinated before but will take it this year_
		0.56_Country.Influ_vaccinationI have not had the vaccination before and will not take it this year_
		0.11_Country.Influ_vaccinationI took it last year and won’t take it this year_
ICC	0.06	
*N*	11_country_	11_county_
Observations	3298	3298

*p < .05, **p < .01, ***p < .001.

Being male increases the likelihood of accepting the theoretical combination by 21% more than being female (OR = 1.21, 95% CI [1.03–1.42], *p* < .05). Having a previous COVID-19 infection decreases the odds of acceptance by 21% more than the reference category (OR = 0.79, 95% CI [0.65–0.95], *p* < .05). Having a family member or friend who died from COVID-19 increases the odds of accepting two vaccinations together than the counterparts with 22% (OR = 1.22, 95% CI [1.03–1.44], *p* < .05). On the continent level, being in Africa increases the acceptance of the theoretical combination by 55% more than the odds of acceptance in Asia.

Additionally, receiving the SIV in the last year and the current year or waiting to take the vaccine this year increases the odds of accepting the vaccine by 60% (95% CI [1.03–2.48]) and 52% (95% CI [1.09–2.13], *p* < .05), respectively. In contrast, losing the intention to take the SIV this year if the respondent didn’t take it last year or took it last year decreased the likelihood of accepting the new theoretical vaccine by 41% (OR = 0.59, 95% CI [0.36–0.98], *p* < .05) and 49% (OR = 0.51, 95% CI [0.33–0.79], *p* < .01, respectively) than the odds of the reference category.

Regarding the status of COVID-19 vaccination, the odds of accepting the theoretical combination if the respondent took the first dose of COVID-19 vaccination and would not take any other doses is 2.29 times greater than the odds of not taking any doses (95% CI [1.62–3.25], *p* < .001). Taking the first and second doses and waiting for the booster dose increased the odds of accepting the theoretical combination by 401% compared to the reference group (OR = 4.01, 95% CI [3.04–5.28], *p* < .05).

The random intercept and slope variances were estimated in the model; they indicate high variability in the effect of the influenza vaccination last year. The variance of the variable categories ranged from 0.05 to 0.56. We added the random slope of the influenza vaccination variable as it showed a significant likelihood ratio test. Still, the rest of the variables didn’t show any significant difference between the variable with only a random intercept and when adding their random slope.

### The goodness of fit measures

To assess the model’s goodness of fit, we compared the model with only a random intercept with the model with a random intercept and a random slope using AIC, BIC and the deviance. We used deviance as a measurement of fit. The lower the deviance, the better the model. The deviance decreased from 4000.3 to 3965.2. The AIC increased trivially from 4060.3 to 4065.2. However, the BIC increased when adding the random slope in the model (from 4243.3 to 4370.2) because of the higher complexity of the model. Additionally, the likelihood ratio test was significant in the models when we added the random slope to the model, assuming a significant difference between models with intercept and models with random intercept and random slopes (*p* < .01962).

## Discussion

The expanding COVID-19 epidemic imposes public health challenges along with the circulation of seasonal influenza viruses. Individuals who didn’t receive any vaccination were more likely to suffer from the COVID-19 infection when compared with those who received both COVID-19 and seasonal influenza vaccines in separate doses. Yet, vaccination non-confidence and hesitancy among the population greatly diminished the vaccine role [28]. Hence, it seems crucial to examine the acceptance rate of the theoretical combination of both vaccines in one shot and to understand factors affecting the public choice of vaccinations to avoid the high rejection rate in the EMR.

In spite of the fact that seasonal influenza is a vaccine-preventable infectious disease, vaccine uptake in general is low in both developed and developing countries [29]. The present research showed that nearly half of the participants (46.3%) neither took it before nor intended to take it this year. About one-fifth of the studied group who did not receive SIV before were willing to get the vaccine this year. In line with this study, the 7th Middle East and North Africa Influenza Stakeholders Network (MENA-ISN) report showed low SIV coverage in the MENA region [30]. Likewise, Ahmed et al. [31] reported that SIV coverage was 11.3% in Yemen. Ghazy et al. [32] also showed a high SIV rejection rate (58.1%) among Libyan respondents. Similarly, a high rate of SIV non-vaccination was high among parents in the EMR as well [33]. Several factors that could hinder better vaccination status include a lack of knowledge about influenza and its consequences, misbeliefs about this vaccine, bad personal experiences and the cost of the vaccination [30].

In studying COVID-19 vaccination, about one-quarter of the respondents didn’t take any doses; 22% completed their vaccination series; 28% were waiting for subsequent doses; and 25.2% stopped vaccinations after the first or second dose. These findings are consistent with previous studies, which reported low vaccine acceptance rates in the EMR towards either the primary series of vaccination or the booster dose [34,35]. This reflects the challenges imposed by COVID-19 vaccine rejection in EMR.

The current work showed that 43.3% of respondents accepted receiving COVID-19 and seasonal influenza vaccinations if available in one shot. Lennon et al. [36] showed that the overall acceptability for the combination of SIV and COVID-19 vaccination was 50% among a nationally representative sample of 12,887 individuals in the United States. They stated that the combination vaccine might increase COVID-19 vaccination coverage. The acceptance rate for the theoretical combination in the current work (43.3%) was higher than that reported in a Libyan study [32]. They showed that only 28.15% of those hesitant about receiving the COVID-19 vaccine would receive it if administered concurrently with the SIV in one shot. The reported reasons for accepting the combination were respondents’ beliefs that a combined vaccine requires fewer doses and was safer, more effective and less costly. Those who rejected the combination claimed that side effects might occur from combining them, and there was no prior evidence of their effects. It is obvious that trust in vaccine safety, effectiveness and efficacy plays a critical role in shaping public attitudes towards vaccination [37].

The present study revealed several predictors for accepting the theoretical vaccine combination, such as socio-demographic characteristics like young age, being male and being African. Similarly, Abrina et al. [38] clarified that several socio-demographic factors were associated with COVID-19 vaccine acceptance. They showed male sex, young age (18–25 years old), being married and working at a government organization were important determinants of high vaccine acceptance. Racial disparities were also highlighted in Ha et al.’s [39] study, with a much higher acceptance rate among white participants (65%) compared to black or African Americans (22%). Inversely, Buzgeia and Abdrabba [40] did not find a relevant association between socio-demographic profile and attitude towards COVID-19 vaccination. Such disparities in vaccine acceptance and uptake highlight the importance of thoroughly examining the factors beyond these observed differences.

Other factors associated with the high acceptance rate of the new vaccine combination were prior COVID-19 vaccination, having a family member died from COVID-19 infection and a positive intention to get the SIV this year. However, respondents who had a previous COVID-19 infection were more likely to reject the combination. These findings are supported by a systematic review and meta-analysis study, which stated that subjects who had previously received the influenza vaccine were more likely to accept the COVID-19 vaccine [41].

On a country level, there was a significant difference between acceptance rates for receiving both vaccines in one dose. More than half of Morocco, Sudan, Afghanistan and Pakistan respondents accepted the two vaccinations in one shot. However, the acceptance rate dropped to 29.7% in Kuwait and 22.3% in Lebanon. The attitude towards vaccines in the EMR region is primarily shaped by many factors, for instance, financial conditions, religious culture, health status, health care services and educational level [42]. High vaccine acceptance rates are usually associated with trust in governmental policies and strategies. In contrast, low vaccine acceptance rates, particularly in low-income countries, are associated with low levels of education and awareness and insufficient governmental efforts [43].

Furthermore, we investigated the relationship between COVID-19 vaccination coverage rates and acceptance of a new theoretical combination. We found that countries with high vaccination coverage rates exhibited low acceptance of the new combination, as observed in Kuwait, Saudi Arabia and Morocco. For instance, on 1 November 2022, during the same period as our study [44], Kuwait reported that 78% of its population had completed the initial vaccination doses. Still, only 29.7% of the studied population in Kuwait accepted the new combination. Conversely, countries with low COVID-19 vaccination coverage rates demonstrated higher acceptance of the new theoretical combination. This trend was evident in countries such as Egypt, Afghanistan, Libya, Iraq, Sudan and Yemen. These findings shed light on a paradoxical relationship between vaccination coverage and acceptance of the new combination.

Other studies have also shown this regional disparity in vaccination coverage and acceptance. Low- and middle-income nations had greater vaccine acceptance but restricted vaccine access, as demonstrated by Solís Arce et al. [45]. High-income countries, on the other hand, are reluctant to receive vaccinations while having more availability to vaccines. This high acceptance rate in less developed countries highlights the importance of equity in vaccine distribution to less developed countries to convert positive intentions into action. This explanation is in line with the theoretical frameworks of the WHO’s Behavioral and Social Drivers of Vaccination model, which suggests that to increase vaccine uptake, we must consider the behavioural and social determinants that influence favourable intentions [46].

Combining seasonal influenza and COVID-19 vaccines may lead to a rise in the uptake of the COVID-19 vaccines, as many populations are already habituated to receiving influenza shots annually, as mentioned by Tzenios et al. [47]. In the same vein, Lennon et al. reported that combining these vaccines offers a practical way to receive two vaccinations, which could lead to a higher rate of COVID-19 vaccination coverage [36]. So, this novel combination could be an effective solution to increase the acceptance of COVID-19 vaccination in countries with high vaccination hesitancy.

### Limitations and strengths

This study has some limitations. First, because of the intrinsic limitations of cross-sectional online surveys, the representativeness of the data may be diminished by sampling bias. Hence, samples collected from countries may not represent the country’s situation. Secondly, the nature of self-reported data can cause recall bias and a tendency to present socially desirable outcomes. Third, the participants joined the study through a non-random sampling method, which may have affected the generalizability of the research. However, this study is unique in exploring the acceptance of combining seasonal influenza and COVID-19 vaccines in one shot to increase vaccine uptake. Additionally, we focused on the real vaccination behaviour after the vaccine became distributed rather than the vaccine intention only. Besides, this study included countries in the EMR based on different income levels reflecting the heterogeneity of the population in the region.

## Conclusions

We can conclude that combining seasonal influenza and COVID-19 vaccines together in one shot increased the overall acceptance of COVID-19 vaccines among vaccine rejectors. The current results also provide reasonable evidence that acceptance of combination vaccines is associated with certain factors, such as socio-demographic profile, prior vaccination history and previous COVID-19 infection. The reasons people accepted this combination were related to safety, effectiveness and cost issues. The main reasons for its rejection were the fear of the side effects and the lack of previous studies about this combination. So, the theoretical combination may be a good solution to overcome the high vaccination hesitancy for any new vaccine.

## Supplementary Material

Supplemental Material

## Data Availability

All data are available upon request from the corresponding author.

## Code availability (software application or custom code)

Data files and R codes are available upon request from the first author.
