# Supplementary material for: Combining COVID-19 and seasonal influenza vaccines together to increase the acceptance of newly developed vaccines in the Eastern Mediterranean Region: a cross-sectional study
Source: Ann Med. 2023 Nov 29;55(2):2286339. doi: 10.1080/07853890.2023.2286339 (PMC10836265; doi:10.1080/07853890.2023.2286339)
Supplement: Supplemental Material [file IANN_A_2286339_SM1256.zip › job categories.docx]

**Job categories**

The participants selected one of these items, then we categorized the responses into four categories “high-skilled (non-manual), low-skilled (non-manual), skilled (manual) and others”. We categorized occupations according to “International Standard Classification of Occupations 2008 (ISCO-08): Structure, group definitions and correspondence tables” Which is published by International Labour Organization (reference 18). (second paragraph of data collection tools page 5-6)

Skill level 1 (others): performing simple and routine physical or manual work. It included also students and non-working participants.

Skill level 2 (low-skilled (non- manual)): performing tasks as operating machinery and electronic equipment.

Skill level 3 and 4 (high-skilled (non-manual)): performing complex technical and practical tasks or performing complex problem-solving, decision-making and creativity.

Skilled manual occupations including craft and related trades workers, skilled agriculture and fishery workers, plant and machine operators and assemblers.

Reference:

International Standard Classification of Occupations 2008 (ISCO-08): Structure, group definitions and correspondence tables. Geneva, Switzerland: International Labour Office; 2012. Available from: https://www.ilo.org/wcmsp5/groups/public/---dgreports/---dcomm/---publ/documents/publication/wcms_172572.pdf
