# Supplementary material for: Combining COVID-19 and seasonal influenza vaccines together to increase the acceptance of newly developed vaccines in the Eastern Mediterranean Region: a cross-sectional study
Source: Ann Med. 2023 Nov 29;55(2):2286339. doi: 10.1080/07853890.2023.2286339 (PMC10836265; doi:10.1080/07853890.2023.2286339)
Supplement: Supplemental Material [file IANN_A_2286339_SM1256.zip › Supplementary Material - Study questionnaire covid infl MENA.docx]

**Combining COVID-19 and seasonal influenza vaccines together to increase the acceptance of newly developed vaccines in the Eastern Mediterranean Region: a cross-sectional study**

**Mohamed Fakhry Hussein ^1^, Abdelhamid Elshabrawy ^2^, Sarah Assem Ibrahim ^3^, Suzan Abdel-Rahman ^4^, Hoda Ali Ahmed Shiba ^5^, Ehab Elrewany ^6^, Mohammad Haroon Hairan^7^, Ramy Mohamed Ghazy ^8^**

This research aims to estimate the acceptance of taking the COVID-19 and seasonal influenza vaccines together and the reasons for accepting or refusing this option. All your information will be confidential and no names will be used when publishing the search. The study was approved by The Ethics Committee of the Faculty of Medicine, Alexandria University, Egypt (IRB number: 00012098). The duration to complete the questionnaire is 5- 10 minutes. You are absolutely free not to participate in the research at any time. Thank you for sharing your valuable time with us. If you agree to register in the search, press Continue.

For any inquiry please contact Dr. Mohamed Fakhry Hussein email: [hiph-mohamedfakhry@alexu.edu.eg](mailto:hiph-mohamedfakhry@alexu.edu.eg)

**I accept to be involved in the research**

- Continue
- Do not want to be in the research

**Chose the language**

- العربية
- English

1. **Respondent socio-demographic data**
2. **Age**

- Less than 18 years
- 18 - less than 25 years
- 25 - less than 35 years
- 35 - less than 50 years
- 50 - 65 years
- Above 65 years

1. **Gender:**

- Male
- Female

1. **Level of education completed**

- Primary education
- Secondary education
- University education
- Post graduated

1. **Marital status**

- Married
- Single
- Widow
- Divorced

1. **Occupation**

- Manager
- Professional job as in medical field or engineer or chemist
- Technicians and Associate Professionals
- Clerical Support Workers
- Service and Sales Workers
- Skilled Agricultural, Forestry and Fishery Workers
- Craft and Related Trades Workers
- Plant and Machine Operators, and Assemblers
- Elementary Occupations
- Armed Forces Occupations
- Student
- Not working / Retired

1. **Nationality**
2. **Now I am living in**
3. **Respondent history of chronic diseases and COVID-19 infection**
4. **Do you suffer from chronic diseases?** conditions that last 1 year or more and require ongoing medical attention or limit activities of daily living or both. Chronic diseases such as heart disease, cancer, renal insufficiency, liver insufficiency, neurological diseases, and diabetes.

- Yes
- No

1. **Have you had COVID-19 infection before?**

- Yes
- No
- I do not know

1. **Do you have any family members/ relatives/ friends who died of COVID-19 infection?**

- Yes
- No
- Maybe

1. **For the  COVID-19  vaccine**
   - I didn't take any doses
   - I only took the first dose and won't take any other doses
   - I took the first dose and am waiting for the second dose
   - I took the first and second doses and waiting for the booster dose
   - I took the first, second and booster doses
   - I took the first and second doses, and I won't take the booster dose
2. **For seasonal Influenza vaccination**
   - I received the vaccination last year
   - I received the vaccination last year and the current year
   - I received the vaccination last year and am waiting for the vaccine this year
   - I didn't get vaccinated before but will take it this year
   - I have not had the vaccination before and will not take it this year
   - I took it last year and won't take it this year

**III. Questions about your attitude towards the theoretical combination between COVID-19 and seasonal Influenza Vaccines together in one shot**

1. **If COVID-19 and seasonal Influenza vaccines are available**
   - I will only take the COVID-19 vaccine
   - I will only take the seasonal Influenza vaccine
   - I will take both vaccines
   - I will not take any of them
2. **If both vaccines (COVID-19 and seasonal Influenza) are available together in one dose**
   - I will take this vaccine
   - I will not take this vaccine
3. **I will take both vaccines (COVID-19 and seasonal Influenza) together in one dose because (you can choose more than one answer)**
   - More safe
   - More effective
   - Less cost
   - Fewer doses
   - Not applicable "I will not take them together or I will not take either of them"
4. **If you prefer to take the two vaccines (COVID-19 and seasonal Influenza) separately, why? (you can choose more than one answer)**
   - Side effects may occur from putting them together
   - It is useless to put them together
   - No studies have been published on their effects together
   - Not applicable "I will take them together or I will not take them"

**Thanks a lot for your time**
